# Post-orthodontic position of lower incisors and gingival recession: A retrospective study

**DOI:** 10.4317/jced.54261

**Published:** 2017-12-01

**Authors:** Domenico Ciavarella, Michele Tepedino, Crescenzio Gallo, Graziano Montaruli, Khrystyna Zhurakivska, Ludovica Coppola, Giuseppe Troiano, Claudio Chimenti, Michele Laurenziello, Lucio Lo Russo

**Affiliations:** 1 Department of Clinical and Experimental Medicine, University of Foggia, Foggia, Italy; 2 Department of Biotechnological and Applied Clinical Sciences, University of L’Aquila, L’Aquila, Italy

## Abstract

**Background:**

To evaluate if changes in lower incisor position following orthodontic treatment are correlated with development of gingival recessions.

**Material and Methods:**

Pre- and post-treatment digital models and lateral cephalograms of 22 subjects were collected retrospectively. The clinical crown length, gingival scallop, and papilla height of the central lower incisor were measured along with the cephalometric incisor’s inclination, the distance from the mandibular plane, and the distance between the Infradentale and Menton points. Statistical correlations between gingival and cephalometric variables were studied. In addition, two groups were defined based on the post-treatment incisor inclination value (‘normal’ or ‘proclined’) and compared.

**Results:**

The incisor inclination was correlated with the change in gingival scallop and papilla height. Moreover, there was a statistically significant difference in clinical crown height and gingival scallop between the ‘normal’ group and the ‘proclined’ group.

**Conclusions:**

Changes in lower incisor position, especially an excessive proclination, after orthodontic treatment may play a role in the development of gingival recession.

** Key words:**Orthodontic treatment, Incisor inclination, IMPA, Gingival recession, Alveolar bone.

## Introduction

The position of the upper and lower incisors is one of the keystones of orthodontic treatment planning. Many authors have pointed out the importance of incisors’ inclination for the aesthetic outcome, since incisors support the lips and determine their prominence and, ultimately, profile attractiveness (1-3). In addition, some important functional aspects like the distribution of masticatory forces, phonation, and condylar guidance are also related to incisors’ inclination (4). It is also commonly accepted that increased protrusion of incisors following orthodontic treatment would be unstable in the long term because of the forces produced by the lips (5,6).

Some authors have observed that excessive proclination of the lower incisors following orthodontic treatment could result in gingival recession. Both animal (7) and human (8-10) studies have observed an apical migration of the gingival attachment as a consequence of labial tooth movement, probably because the teeth were moved out of the alveolar envelope. These findings are not surprising, considering that studies on Cone-Beam Computed Tomography have reported that alveolar fenestrations and dehiscences can be found in about 3% and 8% of the population, respectively (11,12).

However, there is no robust and concurrent scientific evidence supporting these observations. Some authors have reported that there was no association between orthodontic treatment and the development of gingival recession after a five-year follow-up, (13,14) and all the systematic reviews on this argument have concluded that scientific evidence is lacking (15,16). Therefore, the aim of the present retrospective study was to evaluate the periodontal outcome of orthodontic treatment by correlating the post-treatment position of the lower incisors with the changes in clinical crown height (CCH) and alveolar bone height.

## Material and Methods

The records of orthodontic patients consecutively treated from January 2014 to April 2017 at the Dental Clinic, University of Foggia, Italy, were retrospectively screened for the following inclusion criteria:

- Age between 9 and 14 years old

- Pre- and post-treatment lateral cephalogram and dental casts

- Non-extractive orthodontic treatment with full fixed multi-bracket appliance

- Negative anamnesis for systemic diseases and any other conditions that can compromise periodontal health.

**Figure 1:**
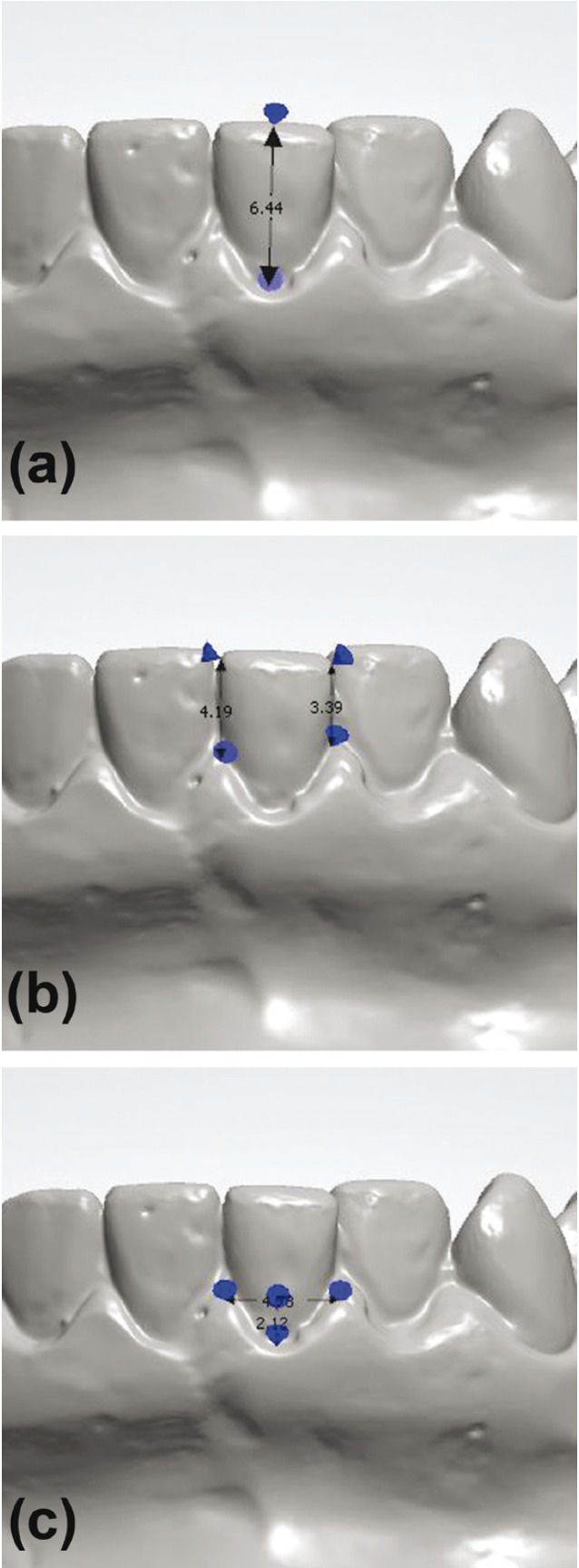
Measurements performed on digital models. (a) Measurement of CCH, the distance between the incisal edge and the gingival zenith; (b) measurement of MPH and DPH, the distance between the gingival papilla and the incisal edge; (c) measurement of GS, the distance between the zenith of the gingival contour and a line connecting the apex of the mesial and distal papilla.

Sample size calculation (G*Power version 3.1.9.2, Universitat Dusseldorf, Germany) (17) for an expected r= 0.6 corresponding to a large effect size (18) revealed a required number of 21 subjects having a first type error of 0.05 and a 90% power. Dental casts taken pre- (T0) and post-treatment (T1) were used to evaluate the changes occurring after incisor proclination in terms of CCH of the lower left central incisor. Post-treatment (T1) dental casts were taken one month after debonding and scaling to overcome the possible influence of bracket-related gingival inflammation on measurements. Dental casts were transformed into digital models using an intraoral scanner (Trios® 3, 3Shape, Copenhagen, Denmark) and then used for further analysis. Ortho Analyzer™ software (Ortho Analyzer™, 3Shape, Copenhagen, Denmark) was used to measure the perpendicular distance between the incisal edge and the most apical point of the free gingival margin (i.e. CCH) for the lower left central incisor. The following variables were measured on the digital models at T0 and T1 (Fig. 1):

- Height of mesial papilla (MPH), the distance (mm) between the incisal edge and the apex of the mesial papilla

- Height of distal papilla (DPH), the distance (mm) between the incisal edge and the apex of the distal papilla

- Gingival scallop (GS), the depth (mm) of the gingival scalloping, measured between the zenith of the gingival contour and a line connecting the apex of the mesial and distal papilla

- CCH, measured (mm) from the incisal edge to the gingival zenith.

**Figure 2:**
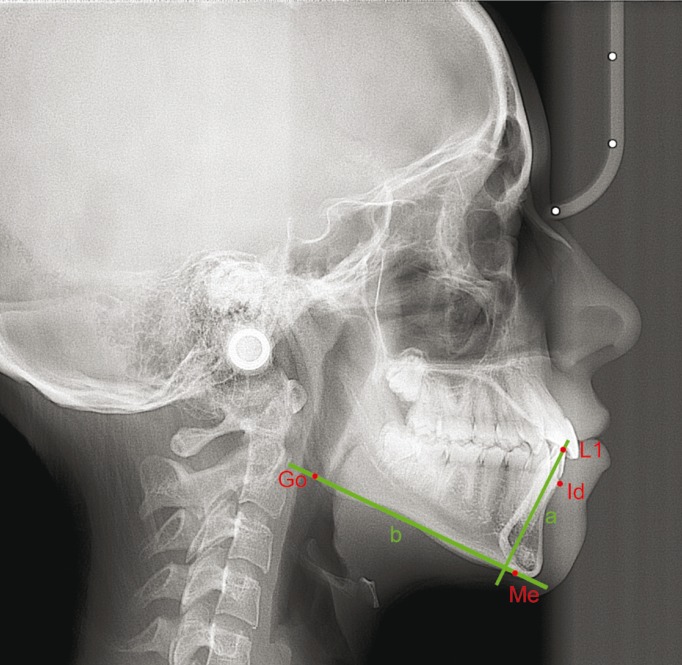
Measurements on lateral cephalograms. L1, incisal edge of the lower central incisor; Id, infradentale point; Me, Menton point; Go, Gonion point; a, long axis of the lower central incisor; b, mandibular plane passing through Go and Me point.

Tracings (FastCeph® version 7, Caes Software srl, Grottaferrata, Italy) on the T0 and T1 lateral cephalograms were used to measure the following values (Fig. 2):

- L1-Me, the perpendicular distance (mm) between the incisal edge of the lower central incisor (L1) and the line parallel to the Frankfurt plane that passes through the Menton (Me) point

- Id-GoMe, the perpendicular distance (mm) between the Infradentale point (the most coronal point of the labial alveolar bone of the mandibular symphysis, Id) and the mandibular plane passing through the Gonion (Go) and Me points

- IMPA, the angle (°) between the long axis of L1 and the mandibular plane passing through the Go and Me points.

For all variables, the difference between the measurements at T1 and T0 were also calculated and reported.

Error of the method

To evaluate the error of the method, all measurements on digital models and lateral cephalograms were repeated after one week by the same operator, and then an intra-class correlation (ICC) coefficient between the two sets of measurements was calculated.

-Statistical analysis

Descriptive statistics for all the variables were calculated, as well as ∆ variables reporting differences between T1 and T0 measurements. The type of distribution for all the variables was then assessed using a Shapiro–Wilk normality test. Further statistical analysis was performed on the T1–T0 differences. Depending on data distribution, a Pearson or a Spearman’s rho correlation test was used to analyse if the post-treatment changes in MPH, DPH, GS, CCH, and alveolar bone height (Id-GoMe) were correlated to the modifications of the lower incisors’ position (L1-Me, IMPA) assessed through the lateral cephalograms. In addition, two groups were defined depending on whether the final IMPA angle at the end of treatment was up to (normal group) or greater than 95° (proclined group), and then the variables defining the gingival contour were compared between the two groups using an independent samples T-test or a Mann–Whitney U-test, depending on data distribution. First type error was set as *p* < 0.05.

## Results

From the records of 60 subjects initially screened, those of 22 patients (7 males and 15 females) were eligible to be included in the study. Mean orthodontic treatment time was three years. Regarding the estimation of the error of the method, the calculated ICC coefficient was excellent (> 0.85) for all the variables, revealing good intra-observer reliability of the measurements.

Descriptive statistics are reported in  Table 1. In general, post-treatment cephalometric variables revealed that L1 was slightly proclined and extruded. Regarding the labial alveolar bone, a small increase in bone height was observed in the mandible. In addition, the CCH decreased after treatment by a small amount, the mesial and distal papilla moved coronally, and the GS deepened ( Table 1).

**Table 1:**
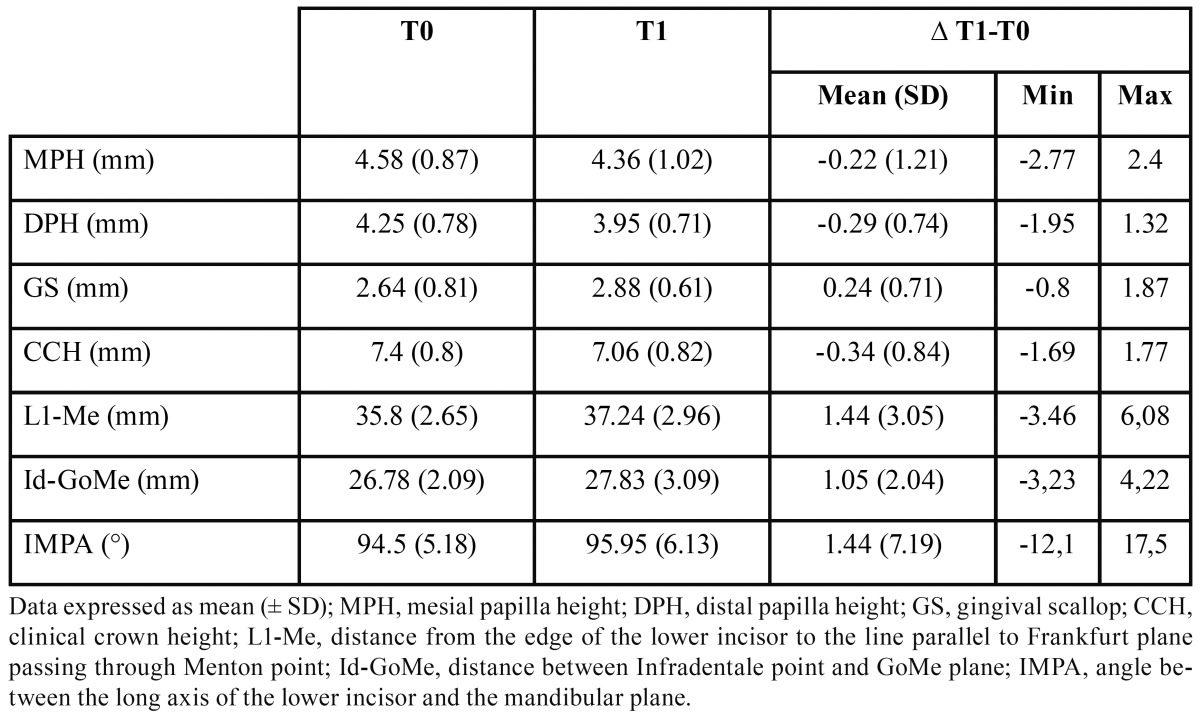
Descriptive statistics for digital model and cephalogram measurements (n = 22).

All variables were normally distributed; therefore, Pearson’s correlation test was used. The IMPA angle variation was negatively correlated with the DPH change and positively correlated with the GS change ( Table 2). On the other hand, the change in labial bone height (∆Id-GoMe) was positively correlated to the increase in the L1–Me distance ( Table 2). An independent samples T-test between the ‘normal’ and ‘proclined’ groups revealed a statistically significant difference between the two groups regarding post-treatment changes of CCH and GS ( Table 3).

**Table 2:**
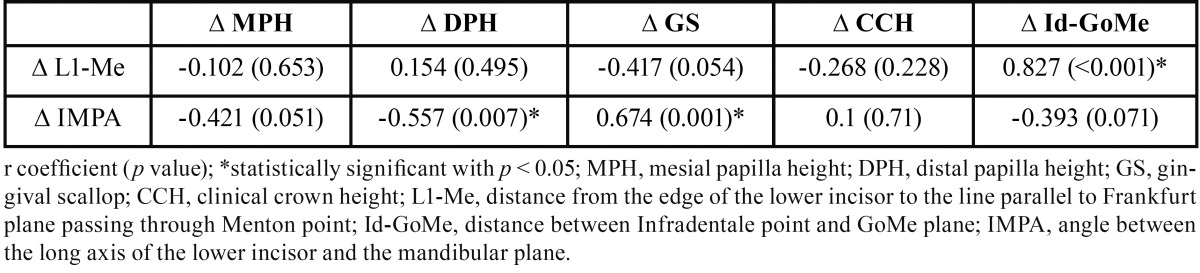
Correlations between gingival and cephalometrics variables (n = 22).

**Table 3:**
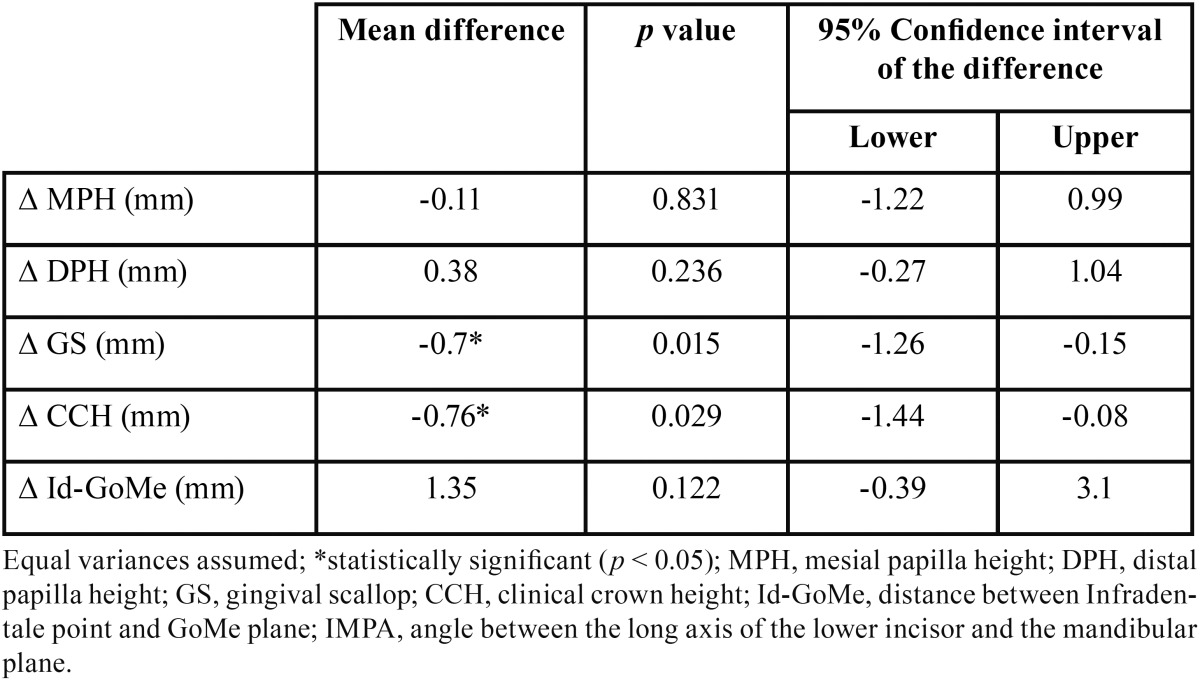
Independent samples T-test between normal (T1 IMPA < 95°, n = 11) and proclined (T1 IMPA > 95°, n = 11) group.

## Discussion

The use of digital models has many advantages over plaster models: handling and storage are easier; measurements are faster; and data can be transferred directly to another electronic database, eliminating possible errors due to manual transcription. In addition, measurements made on plaster models and digital models were proved to be equally accurate (19,20), whereas other authors found digital models even more accurate and reproducible (21). This was confirmed by the excellent intra-rater agreement found in the present study.

The post-treatment changes of the position of the lower central incisors, as reported in  Table 1, revealed that orthodontic treatment resulted by mean in a slight proclination of the lower incisors, even though the large standard deviations suggest that the outcomes were variable among all the cases. Indeed, dividing the sample into two groups depending on the post-treatment IMPA value revealed that 11 subjects had normally inclined incisors (IMPA < 95°) and 11 subjects had proclined (IMPA > 95°) incisors ( Table 3). In addition, the lower incisors slightly extruded. The labial inclination movement of the tooth also produces a downward displacement of the incisal edge; therefore, the real extrusion movement was greater than the one measured ( Table 1). The extrusive movement of the lower incisor was followed by a similar increase in alveolar bone height, showing a statistically significant (*p* < 0.001) correlation ( Table 2). This finding can probably be related to both orthodontic biomechanics and the vertical growth of the alveolar process. No significant difference in alveolar bone level (∆Id-GoMe) was observed between the ‘normal’ and ‘proclined’ groups ( Table 3); however, the true three-dimensional anatomy of the bone cannot be appreciated via lateral cephalograms.

On the other hand, the soft tissues moved coronally: the clinical crown length was reduced after treatment and the apex of the mesial and distal papilla was displaced coronally; consequently, the GS deepened after treatment. The post-treatment measurements were taken one month after debonding and a scaling session to avoid the possible influence of gingival swallowing and inflammation caused by the hygiene impairment provided by the orthodontic fixed appliance. Therefore, the measured gingival increase was not influenced by tissue swallowing, and it could be explained also by the alveolar bone that migrated coronally. The difference in DPH was negatively correlated with the change in the IMPA angle, whereas the difference in GS was positively correlated. The variation in MPH also showed a good correlation (r = -0.421) but was not statistically significant (*p* = 0.051).

To deeply investigate the influence of the post-treatment incisor position (IMPA) on the gingival margin, the sample was divided into ‘normal’ and ‘proclined’ groups based on the post-treatment IMPA value. Comparison of the post-treatment variation in the gingival profile between those two groups revealed that the gingival margin moved apically in the proclined group by nearly 0.7 mm ( Table 3), and this difference was statistically significant (*p* < 0.05). This finding is interesting because it supports the idea that the final inclination of the lower incisors, rather than the orthodontic treatment by itself, can lead to gingival recession (22).

Bony dehiscence and fenestration are common findings, especially in the mandible, (23) and the orthodontic proclination of the lower incisors may further reduce the quantity of bone surrounding the roots and thin down the gingival margin, thus increasing the risk of gingival recession. This theory was confirmed in animal models, where after proclination a proportional marginal bone loss was observed (24,25). Similar results in humans are not yet clearly demonstrated; some studies using CBCT imaging have observed that thinner mandibular symphysis and thinner pre-treatment cortical bone were correlated with a higher risk of post-treatment labial bone loss, but this was not correlated with IMPA and incisor position (26,27). Part of the literature negates strong evidence that the incisors’ proclination following orthodontic treatment results in gingival recessions (13,15,16,28), suggesting that local host factors like gingival biotype, soft tissue quality, and alveolar bone thickness may play a major role in the development of gingival recession.

One of the limitations of the present study was the short observation period. However, over a long observation time many other factors can be introduced and act as confounders. Many factors concur in the development of gingival recession, like oral hygiene, brushing habits, and smoking, which would be difficult to control and isolate. Moreover, some findings suggest that gingival recession following orthodontic treatment is not progressive. For these reasons, high-quality prospective or randomised studies on this argument are lacking; therefore, no robust scientific evidence exists. In addition, it would be challenging to randomise groups of patients with different types of incisor movements. In such a case, non-randomised observational studies can be helpful in providing evidence and guidelines for future studies (29-31).

## Conclusions

Considering the findings of the present study and the uncertainty of data found in the literature, the clinical advice that can be taken is to be conservative and respect the alveolar bone envelope, avoiding excessive proclination of the lower incisors. In conclusion, within the limitations of the present study, post-orthodontic treatment evaluation revealed that modification of the gingival contour of the lower incisor was correlated with a change in incisor position. Patients whose lower incisors were proclined more than 95° at the end of orthodontic treatment showed an apical migration of the gingival zenith compared to those subjects who had a normal incisor inclination.

## References

[1] Margolis HI (1943). The Axial Inclination of the Mandibular Incisors. Am J Orthod Oral Surg.

[2] Downs WB (1956). Analysis of the Dentofacial Profile. Angle Orthod.

[3] Ricketts RM (1960). Cephalometric synthesis. Am J Orthod.

[4] Silness J, Johannessen G, Røynstrand T (1993). Longitudinal relationship between incisal occlusion and incisal tooth wear. Acta Odontol Scand.

[5] Artun J, Krogstad O, Little RM (1990). Stability of mandibular incisors following excwssive proclination: a study in adults with surgically treated mandibular prognathism. Angle Orthod.

[6] Little RM (2002). Stability and relapse: Early treatment of arch length deficiency. Am J Orthod Dentofacial Orthop.

[7] Wennstrom JL, Lindhe J, Sinclair F, Thilander B (1987). Some periodontal tissue reactions to orthodontic tooth movement in monkeys. J Clin Periodontol.

[8] Geiger AM (1980). Mucogingival problems and the movement of mandibular incisors: A clinical review. Am J Orthod.

[9] Coatoam GW, Behrents RG, Bissada NF (1981). The width of keratinized gingiva during orthodontic treatment: its significance and impact on periodontal status. J Periodontol.

[10] Boyd RL (1978). Mucogingival Considerations and Their Relationship to Orthodontics. J Periodontol.

[11] Enhos S, Uysal T, Yagci A, Velid I, Ucare FI, Ozerf T (2012). Dehiscence and fenestration in patients with different vertical growth patterns assessed with cone-beam computed tomography. Angle Orthod.

[12] Rupprecht RD, Horning GM, Nicoll BK, Cohen ME (2001). Prevalence of Dehiscences and Fenestrations in Modern American Skulls. J Periodontol.

[13] Renkema AM, Navratilova Z, Mazurova K, Katsaros C, Fudalej PS (2015). Gingival labial recessions and the post-treatment proclination of mandibular incisors. Eur J Orthod.

[14] Renkema AM, Fudalej PS, Renkema A, Bronkhorst E, Katsaros C (2013). Gingival recessions and the change of inclination of mandibular incisors during orthodontic treatment. Eur J Orthod.

[15] Aziz T, Flores-Mir C (2011). A systematic review of the association between appliance-induced labial movement of mandibular incisors and gingival recession. Aust Orthod J.

[16] Joss-Vassalli I, Grebenstein C, Topouzelis N, Sculean A, Katsaros C (2010). Orthodontic therapy and gingival recession: a systematic review. Orthod Craniofac Res.

[17] Faul F, Erdfelder E, Lang A G, Buchner A (2007). G*Power: A flexible statistical power analysis program for the social, behavioral, and biomedical sciences. Behav Res Methods.

[18] Cohen J (1992). A Power Primer. Psychol Bull.

[19] Dória Cabral Correia G, Habib FAL, Vogel CJ (2014). Tooth-size discrepancy : A comparison between manual and digital methods. Dental Press J Orthod.

[20] Bootvong K, Liu Z, McGrath C, Hägg U, Wong RWK, Bendeus M (2010). Virtual model analysis as an alternative approach to plaster model analysis: Reliability and validity. Eur J Orthod.

[21] Dowling AH, Burns A, Macauley D, Garvey TM, Fleming GJP (2013). Can the intra-examiner variability of Little's Irregularity Index be improved using 3D digital models of study casts?. J Dent.

[22] Yared KFG, Zenobio EG, Pacheco W (2006). Periodontal status of mandibular central incisors after orthodontic proclination in adults. Am J Orthod Dentofac Orthop.

[23] Yagci A, Veli I, Uysal T, Ucar FI, Ozer T, Enhos S (2012). Dehiscence and fenestration in skeletal Class I, II, and III malocclusions assessed with cone-beam computed tomography. Angle Orthod.

[24] Steiner GG, Pearson JK, Ainamo J (1981). Changes of the Marginal Periodontium as a Result of Labial Tooth Movement in Monkeys. J Periodontol.

[25] Batenhorst KF, Bowers GM, Williams JE (1974). Tissue Changes Resulting from Facial Tipping and Extrusion of Incisors in Monkeys. J Periodontol.

[26] Garlock DT, Buschang PH, Araujo EA, Behrents RG, Kim KB (2016). Evaluation of marginal alveolar bone in the anterior mandible with pretreatment and posttreatment computed tomography in nonextraction patients. Am J Orthod Dentofacial Orthop.

[27] Sun B, Tang J, Xiao P, Ding Y (2015). Presurgical orthodontic decompensation alters alveolar bone condition around mandibular incisors in adults with skeletal Class III malocclusion. Int J Clin Exp Med.

[28] Renkema AM, Fudalej PS, Renkema AAP, Abbas F, Bronkhorst E, Katsaros C (2013). Gingival labial recessions in orthodontically treated and untreated individuals: a case - control study. J Clin Periodontol.

[29] Shrier I, Boivin JF, Steele RJ, Platt RW, Furlan A, Kakuma R (2007). Should meta-analyses of interventions include observational studies in addition to randomized controlled trials? A critical examination of underlying principles. Am J Epidemiol.

[30]  Reeves  B,  Deeks  J,  Higgins  J,  Wells  G (2008). . Including non- randomized studies. In: Cochrane handbook for systematic reviews of interventions.

[31] Pandis N (2011). The evidence pyramid and introduction to randomized controlled trials. Am J Orthod Dentofac Orthop.

